# Supplementary material for: Intersect between brain mechanisms of conditioned threat, active avoidance, and reward
Source: Commun Psychol. 2025 Feb 26;3:32. doi: 10.1038/s44271-025-00197-7 (PMC11864974; doi:10.1038/s44271-025-00197-7)
Supplement: Supplementary file 2 — Reporting summary [file 44271_2025_197_MOESM2_ESM.pdf]

nature portfolio

Corresponding author(s): Mohammed R. Milad

Last updated by author(s): Dec 20, 2024

Reporting Summary

Nature Portfolio wishes to improve the reproducibility of the work that we publish. This form provides structure for consistency and transparency in reporting. For further information on Nature Portfolio policies, see our Editorial Policies and the Editorial Policy Checklist.

Statistics

For all statistical analyses, confirm that the following items are present in the figure legend, table legend, main text, or Methods section.

n/a	Confirmed	
☒	☐	The exact sample size (*n*) for each experimental group/condition, given as a discrete number and unit of measurement
☒	☐	A statement on whether measurements were taken from distinct samples or whether the same sample was measured repeatedly
☒	☐	The statistical test(s) used AND whether they are one- or two-sided<br>*Only common tests should be described solely by name; describe more complex techniques in the Methods section.*
☒	☐	A description of all covariates tested
☒	☐	A description of any assumptions or corrections, such as tests of normality and adjustment for multiple comparisons
☒	☐	A full description of the statistical parameters including central tendency (e.g. means) or other basic estimates (e.g. regression coefficient) AND variation (e.g. standard deviation) or associated estimates of uncertainty (e.g. confidence intervals)
☒	☐	For null hypothesis testing, the test statistic (e.g. *F*, *t*, *r*) with confidence intervals, effect sizes, degrees of freedom and *P* value noted<br>*Give P values as exact values whenever suitable.*
☒	☐	For Bayesian analysis, information on the choice of priors and Markov chain Monte Carlo settings
☒	☐	For hierarchical and complex designs, identification of the appropriate level for tests and full reporting of outcomes
☒	☐	Estimates of effect sizes (e.g. Cohen's *d*, Pearson's *r*), indicating how they were calculated

Our web collection on statistics for biologists contains articles on many of the points above.

Software and code

Policy information about availability of computer code

Data collection	We did not collected primary data, so no software or code was used for this purpose.
Data analysis	We synthesized the findings from existing studies to build a model about avoidance, threat and reward.<br>We used Mendeley Reference Manager 2.100.0 to manage and organize the references.<br>We used BioRender platform (BioRender.com) and GraphPad Prism 10.2.1 to generate the Figures.

For manuscripts utilizing custom algorithms or software that are central to the research but not yet described in published literature, software must be made available to editors and reviewers. We strongly encourage code deposition in a community repository (e.g. GitHub). See the Nature Portfolio guidelines for submitting code & software for further information.

Data

Policy information about availability of data

All manuscripts must include a data availability statement. This statement should provide the following information, where applicable:

- Accession codes, unique identifiers, or web links for publicly available datasets
- A description of any restrictions on data availability
- For clinical datasets or third party data, please ensure that the statement adheres to our policy

The prospective paper does not include primary data collection. All data discussed in the paper was previously published and cited in the references list. All papers are accessible through public databases, such as PubMed and Google Scholar.

Research involving human participants, their data, or biological material

Policy information about studies with human participants or human data. See also policy information about sex, gender (identity/presentation), and sexual orientation and race, ethnicity and racism.

Reporting on sex and gender	The focus of this prospective paper was beyond sex and gender. Where applicable, any relevant mentions of sex or gender in the cited studies are included in the discussion. Still, this was not the primary focus of the paper.
Reporting on race, ethnicity, or other socially relevant groupings	The paper does not directly dresses race or ethnicity as we synthesized model focuses on avoidance, threat and reward. Any mention of these background factors in the previously published papers were mentioned in our manuscript when relevant. But this was not the main focus of this prospective study.
Population characteristics	This prospective paper included findings from published papers conducted on rodents and humans (with and without psychopathology). We included translational aspects of avoidance and discussed mechanisms of adaptive vs. maladaptive avoidance.
Recruitment	We did not involved participants recruitment. Details about recruitment are reported in the original publications listed in the references of this paper.<br>All papers that we used in the study are available in academic publishers and public databases, such as PubMed.
Ethics oversight	As this is a prospective paper with no collection of original data or interaction with participants, ethical approval was not required. The ethical considerations of the cited papers are outlined on the original studied.

Note that full information on the approval of the study protocol must also be provided in the manuscript.

Field-specific reporting

Please select the one below that is the best fit for your research. If you are not sure, read the appropriate sections before making your selection.

☐ Life sciences ☒ Behavioural & social sciences ☐ Ecological, evolutionary & environmental sciences

For a reference copy of the document with all sections, see nature.com/documents/nr-reporting-summary-flat.pdf

Behavioural & social sciences study design

All studies must disclose on these points even when the disclosure is negative.

Study description	This is a perspective study used previously published papers to integrate a neurobehavioral model of avoidance, threat and reward.
Research sample	No direct research participants or sampling was involved. We used the insights of published papers on the field of avoidance.
Sampling strategy	We selected the published papers based on their relevance to avoidance. As this is not a systematic review paper, no formal sampling strategy was applied.
Data collection	This study does not include primary data collection. We derived information from previously published studies, accessed through academic databases, such as PubMed, Google Scholar, etc. We extract key findings from the previously published papers to synthesize a new model for avoidance, threat and reward.
Timing	The majority of the literature that we used in the paper was published between 2010-2024.
Data exclusions	As this is not a systematic review paper, we did not employ any predefined exclusion criteria. Studies were excluded informally if the focus was outside this paper scope. This process was guided by the authors' expertise in the field.
Non-participation	We did not involve participants or data collection. No participants were enrolled or dropped from this study.
Randomization	The reviewed articles were selected based on relevance to avoidance. Randomization was not applicable in this case.

Reporting for specific materials, systems and methods

We require information from authors about some types of materials, experimental systems and methods used in many studies. Here, indicate whether each material, system or method listed is relevant to your study. If you are not sure if a list item applies to your research, read the appropriate section before selecting a response.

Materials & experimental systems

n/a	Involved in the study
☒	☐ Antibodies
☒	☐ Eukaryotic cell lines
☒	☐ Palaeontology and archaeology
☒	☐ Animals and other organisms
☒	☐ Clinical data
☒	☐ Dual use research of concern
☒	☐ Plants

Methods

n/a	Involved in the study
☒	☐ ChIP-seq
☒	☐ Flow cytometry
☒	☐ MRI-based neuroimaging

Plants

Seed stocks

Seeds and plants are out of our field.

Novel plant genotypes

This is a psychology study. We did not discuss any plant work.

Authentication

This is out of our expertises. We focus on behavioral neuroscience rather than plants.
